# Assessment of changes in place of death of older adults who died from dementia in the United States, 2000–2014: a time-series cross-sectional analysis

**DOI:** 10.1186/s12889-020-08894-0

**Published:** 2020-06-11

**Authors:** Wei Xu, Changshan Wu, Jason Fletcher

**Affiliations:** 1 Center for Demography of Health and Aging, University of Wisconsin – Madison, Madison, WI USA; 2 Department of Geography, University of Wisconsin – Milwaukee, Milwaukee, WI USA; 3 La Follette School of Public Affairs, Departments of Sociology, Agricultural and Applied Economics, and Population Health Sciences, University of Wisconsin – Madison, Madison, WI USA

**Keywords:** Dementia, Place of death, End-of-life care

## Abstract

**Background:**

As the mortality attributable to dementia-related diseases in the United States escalates, providing quality and equitable end-of-life care for dementia patients across care settings has become a major public health challenge. Previous research suggests that place of death may be an indicator of quality of end-of-life care. This study aims to examine the geographical variations and temporal trends in place of death of dementia decedents in the US and the relationships between place of death of dementia decedents and broad structural determinants.

**Methods:**

Using nationwide death certificates between 2000 and 2014, we described the changes in place of death of dementia decedents across states and over time. Chi-square test for trend in proportions was used to test significant linear trend in the proportion of dementia decedents at difference places. State fixed effects models were estimated to assess the relationships between the proportion of dementia decedents at difference places and state-level factors, particularly availability of care facility resources and public health insurance expenditures.

**Results:**

Dementia decedents were more likely to die at home and other places and less likely to die at institutional settings over the study period. There was wide inter-state and temporal variability in the proportions of deaths at different places. Among state-level factors, availability of nursing home beds was positively associated with rates of nursing home/long term care deaths and negatively associated with rates of home deaths. Medicaid expenditure on institutional long term supports and services was positively associated with rates of nursing home/long term care deaths and negatively associated with rates of home deaths. Medicaid expenditure on home and community based services, however, had a positive association with rates of home deaths.

**Conclusions:**

There was a persistent shift in the place of death of dementia decedents from institutions to homes and communities. Increased investments in home and community based health services may help dementia patients to die at their homes. As home becomes an increasingly common place of death of dementia patients, it is critical to monitor the quality of end-of-life care at this setting.

## Background

Dementia is a major contributor to mortality in the US. According to the National Center for Health Statistics (NCHS), Alzheimer’s disease (AD), the most common form of dementia, was the underlying cause of 121,404 deaths and the 6th leading cause of death in the US in 2017 [1]. The age-standardized AD mortality rate increased from 18.0 per 100,000 persons in 2000 to 31.0 in 2017 [2, 3]. In 2050, 1,600,000 US older adults will die with AD dementia, nearly triple the number in 2010 [4]. The number of dementia-related deaths will be even greater if other subtypes of dementia are included. This increasing trend of dementia mortality poses a significant challenge for the federal and state health care systems to adequately and efficiently provide quality care for dementia patients toward the end of their lives [5].

Current evidence suggests that place of death may be an indicator of quality of end-of-life care. It is generally reported that people dying at home (especially with hospice services) had better quality of life, experienced fewer symptoms and less discomfort, and their caregivers were less likely to develop psychiatric illnesses [6–8]. We did not find any empirical studies of dementia patients’ preference for place of death. However, for persons with other terminal illnesses or the general population, most people prefer home death, regardless of cultural settings [9–14]. For many dementia patients in the US, dying at home is still a distant reality. Examining spatial and temporal patterns in place of death of dementia decedents in the US may inform health policies aimed to optimize the organization and delivery of palliative care across settings and facilitate home death for dementia patients.

Existing studies on place of death in the US predominately focused on all-cause [15–18] or cancer-specific [6, 18–22] deaths, with only a few exceptions on deaths from a dementia-related disease. For instance, a study found that among Medicaid-eligible persons with dementia in the South Carolina Alzheimer’s Disease Registry between 1988 and 1994, 8% died at home, 13% died at mental health nursing facilities, 27% died in nursing homes, and 51% died in hospitals (including hospice) [23]. A national study of US older persons whose underlying cause of death was dementia showed that in 2001, the most common place of death was nursing home (66.9%), followed by hospital (15.6%), home (12.7%) and other (4.7%). There was wide state-to-state variability in terms of where dementia decedents died [24]. Studies comparing place of death from dementia and other major leading causes of death in the US found that persons who died from dementia were much more likely to die at nursing home than those who died from cancer or other conditions [24, 25].

The distributions of place of dementias-related deaths in European countries were quite different. A study in Finland found that primary care hospital (39.8%) (public sector facilities such as community hospitals play a major role in providing primary care in Finland) was the most common place of death among people with dementia in 2013, followed by sheltered housing with 24-h assistance (24.7%), nursing home (20.8%), and home (8.1%). Between 1998 and 2013, dying at primary care hospitals decreased while dying at sheltered housing with 24-h assistance increased [26]. In Belgium, 58.2% of dementia patients died at care home in 2008, compared to 24.6% at hospital, 13.4% at home, and 3.0% at palliative care unit [27]. In England, hospital was the most common place of death among people who died with dementia between 2000 and 2010; however, the trend towards increasing hospital death of dementia patients reversed mid-decade and an increasing proportion of deaths occurred in care homes [28]. In a study of five European countries (Belgium, the Netherlands, England, Scotland, and Wales), it was found that between 50% (Wales) and 92% (Netherlands) of patients with dementia died in a nursing home and between 3% (Netherlands) and 46% (Wales) died in a hospital. Home death was rare (3–5%), except in Belgium (11%) [5]. In another study of international variations in place of death of older people who died from dementia in 14 European and non-European countries, the authors found substantial differences in the frequency distributions of places of death (proportion of home deaths: from 3.4% in Canada to 69.3% in Mexico; hospital deaths: from 1.6% in the Netherlands to 73.6% in South Korea; long term care setting: from 5.5% in South Korea to 93.1% in the Netherlands) [29].

Place of death may be influenced by a multitude of factors, including age [24, 28–31], sex/gender [28–31], race/ethnicity [32], marital status [28, 29], education level [29], informal carer support [14], hospice enrollment [30], general practitioner’s awareness of patient preference [33], patient-family caregiver congruence on preferred place of death [34], urbanization of place of residence [28, 29], area deprivation [28], and availability of health care resources (e.g. general practitioners and hospital/nursing home bed) [24, 28–30]. Other factors such as nature/severity of terminal illnesses and comorbidities may also play a role. Although previous studies have shed light on the place of death of dementia decedents in the US, a few knowledge gaps remain: How has the place of death of dementia decedents in the US changed over time? To what extent do states differ in terms of the changes? What are the relationships between place of death of dementia decedents and broader structural factors (e.g. access to health care resources, public care program investments)? These questions will have major implications for the organization of end-of-life care for dementia patients in different care settings. To bridge these gaps, we used nationwide death certificates between 2000 and 2014 to examine the geographical variations and temporal trends in the place of death of US older adults who died from dementia and to relate proportions of dementia deaths at different places to state level factors. This study aimed to address following questions:

1. What is the frequency distribution of place of death among older adults who died from a dementia-related disease in the US? How has it changed over time?
2. What are the inter-state variations in such changes?
3. What are the associations between place of death of dementia decedents and characteristics of care facility provision and care financing at the state level?

## Methods

### Data

The data used in this study were derived from all individual death certificates completed between 2000 and 2014 in the US [35]. Identification of records of deaths from dementia was based on the *underlying cause of death*, which is defined as “the disease or injury that initiated the train of morbid events leading directly to death or the circumstances of the accident or violence that produced the injury” [36]. The cause of death on death certificates after 1999 was coded according to the *International Classification of Diseases, Tenth Revision* (ICD-10) [37]. For our analysis, older adults (persons aged 65 years and older) with a dementia-related disease (ICD-10 codes: F01 (vascular dementia), F02 (Dementia in other diseases classified elsewhere), F03 (unspecified dementia), G30 (Alzheimer’s disease)) recorded as the underlying cause of death were selected as the study sample. Persons with foreign and US territory residential status were excluded.

### Variables

The outcome variable is state- and year-specific proportion of dementia deaths at a certain place of death among all dementia deaths in that state and year. Values of *Place of Death and Decedent’s Status* field on death certificates were recoded into four categories: Decedent’s home, Nursing home/long term care, Hospital (includes hospital, clinic, and medical center), and Other^1^ (includes hospice facility, other, and place of death unknown). Socio-demographic characteristics (sex, race/ethnicity, age at death, marital status, and educational attainment), state of residence and year of death of decedents were also extracted to construct the outcome variable and examine the disparities in place of death between different population cohorts and states.

Three major components were used as independent variables to examine the associations between place of death of US older adults who died from dementia and state-level factors:

1. *Socio-demographic structure of dementia decedents.* The structure was measured by variables including state- and year-specific percentage of decedents aged 85 years and older (AGE), percentage of decedents who were female (FEMALE), percentage of decedents who were non-Hispanic White (WHITE), percentage of decedents who were married (MARRIED), and percentage of decedents who had high school or less educational attainment (EDUC). Values of these variables were generated from the death certificates data.
2. *Care facility resources*. We used two variables to indicate the availability of care facility recourses, including state- and year-specific number of hospital beds (HB) per 1000 population and number of nursing home beds (NHB) per 1000 older adults. The number of hospital beds per 1000 population were generated by Henry J Kaiser Family Foundation with the American Hospital Association Annual Survey data [38]. The number of nursing home beds by state and year were obtained from *Health, United States*, an annual report by the NCHS on trends in health statistics [39]. Census and intercensal estimates of state population by age groups were used to calculate the number of nursing home beds per 1000 older adults for each state across the years [40, 41].
3. *Public care financing*. We used price-, age-, sex-, and race-adjusted Medicare (Part A and Part B) reimbursement rates, including Continuous Medicare History Sample (CMHS)- or claim-based home health agencies (HHA) reimbursement per enrollee in 1000 dollars and CMHS- or claim-based hospital and skilled nursing facility (HSNF) reimbursement per enrollee in 1000 dollars,^2^ to measure Medicare benefit spending in care services that might be utilized by dementia patients. In traditional Medicare, health care providers file claims for reimbursement to Medicare for services delivered to Medicare beneficiaries. The rate adjustment accounts for differences in Medicare spending as a results of state differences in prices of services and service utilization due to differences in population composition. Medicaid expenditures variables included state- and year-specific total federal and state Medicaid expenditure on institutional long term services and supports (LTSS) and on home and community based services (HCBS). Data on state- and year-specific Medicare reimbursement rates and Medicaid expenditures were obtained from The Dartmouth Atlas of Health Care [42]*.* State population mentioned above were also used to calculate state- and year-specific Medicaid expenditures on institutional LTSS and HCBS per older adults.

### Statistical analysis

Dementia decedents’ socio-demographic characteristics by place of death were described using percentages. Dot plots were used to visualize the inter-state variations and temporal changes in the proportions of dementia deaths at different places between 2000 and 2014. State fixed effects regression models were used to examine a wide range of state level factors with respect to their abilities to explain variations in the place of death of dementia patients. By exclusively using within-state comparisons, state fixed effects models account for state heterogeneity due to unobserved time-invariant variables that may confound the estimates. In this case, coefficient estimate of an independent variable can be interpreted as the average change in the dependent variable as the independent variable increases by one-unit *over time* while other independent variables are held constant [43, 44]. For a panel data set of 43 states and 15 years, the state fixed effects models can be specified as following:

1 $$ {\displaystyle \begin{array}{l}\mathrm{logit}\left({\mathit{\Pr}}_{it}\right)={\beta}_0+{\beta}_1{AGE}_{it}+{\beta}_2\mathrm{F}{EMALE}_{it}++{\beta}_3{WHITE}_{it}+{\beta}_4{MARRIED}_{it}\\ {}+{\beta}_5{EDUC}_{it}+{\beta}_6{HB}_{it}+{\beta}_7{NHB}_{it}+{\beta}_8{HHA}_{it}+{\beta}_9{HSNF}_{it}\\ {}+{\beta}_{10} Institutial{LTSS}_{it}+{\beta}_{11}{HCBS}_{it}+{c}_i+{\varepsilon}_{it}\end{array}} $$

where, for *i* = 1, …, 43 and *t* = 1, …, 15, *Pr*_*it*_ is the proportion of dementia deaths at a certain place for state *i* in year *t*, *β*_0_ is the intercept term, *β*_1_…*β*_11_ are the coefficients of predictors, *c*_*i*_ is the time-invariant state-specific intercept which can be understood as state fixed effects, and *ε*_*it*_ is the random error term assumed to be independently and identically distributed. Since *Pr*_*it*_ is a proportion, using it directly as dependent variable could result in the problem that the predicted value falling outside [0, 1]. To mitigate this issue, the dependent variable was logit-transformed. As robustness check, we estimated state and time two-way fixed effects models to additionally account for unobserved confounders (e.g. common shocks) that are fixed across states but vary over time. For the ease of interpretation and suitability for our research questions [43], we chose to present the results from state fixed effects models in the main article. Statistical tests were two-sided and a *p*-value of less than 0.05 indicated that the coefficient estimate was statistically significant. Analyses were performed using the R package “*plm”* [45] in RStudio.

## Results

### National trends

**Fig. 1:**
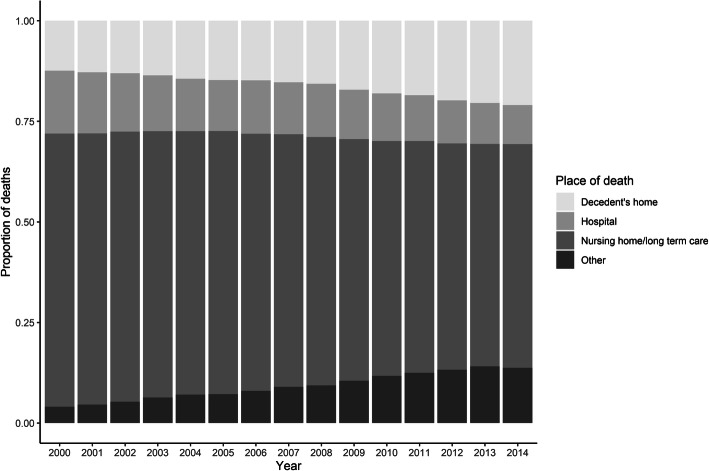
National trends in the place of death with dementia as the underlying cause among older adults in the United States, 2000–2014

Seventy-five thousand four hundred forty-two older adults died from dementia as the underlying cause in 2000, of which 9375 (12.4%) died at decedent’s home, 11,801 (15.6%) died at hospital, 51,209 (67.9%) died at nursing home/long term care, and 3057 (4.1%) died at other places. In 2014, 223,011 older adults died from dementia as the underlying cause, of which 46,802 (21.0%) died at decedent’s home, 21,626 (9.7%) died at hospital, 123,981 (55.6%) died at nursing home/long term care, and 30,602 (13.7%) died at other places. Figure 1 shows the changes in the yearly proportions of deaths at different places during the study period. Nationally, there was a steady increase in the proportions of deaths at decedent’s home and other places; while the proportions of deaths at institutional settings such as nursing home/long term care and hospital decreased. Chi-squared (χ2) testing for trend in proportions showed that there was a significant linear trend in the proportions of deaths at each of the four places of death over the years (*p* < 0.01 for all four categories), indicating persistent structural changes in the place of death of dementia decedents during the study period.

Table 1 shows the numbers and proportion of dementia deaths occurred at different places of death in 2000 and 2014, in all older adult decedents and different socio-demographic cohorts. In 2000, the proportion of nursing home/long term care deaths was much higher in females compared to in males (Female: 70.8% vs. Male: 60.9%); while the proportion of hospital deaths in females was much lower (Female: 12.8% vs. Male: 22.6%). Proportions of deaths at decedent’s home and other places did not differ substantially by sex. Compared with other racial/ethnic groups, non-Hispanic Whites were less likely to die at home and hospital and much more likely to die at nursing home/long term care facilities (approximately 7 out of 10 non-Hispanic Whites versus less than half for other race/ethnicity groups). Hispanics had the highest proportion of home deaths (24.9%) while non-Hispanic Blacks had the highest proportion of hospital deaths (31.8%). Decedents who died at younger ages were more likely to die at home and hospital; while those who died at older ages were increasingly more likely to die at nursing home/long term care settings. Compared to decedents who were single or divorced, those who were married at time of death were more likely to die at their home and hospital and less likely to die at nursing home/long term care facilities. In terms of education attainment, those who were more educated were more likely to die at home and less likely to die at nursing home/long term care facilities.

**Table 1:** Distribution of place of death for US older adults who died from dementia in 2000 and 2014, n(%)

	2000				2014			
	Decedent’s home	Hospital	Nursing home / long term care	Other	Decedent’s home	Hospital	Nursing home / long term care	Other
**All deaths**	9375 (12.4)	11,801 (15.6)	51,209 (67.9)	3057 (4.1)	46,802 (21.0)	21,626 (9.7)	123,981 (55.6)	30,602 (13.7)
**Sex**								
Male	2704 (12.3)	4949 (22.6)	13,341 (60.9)	925 (4.2)	14,894 (21.3)	9106 (13.0)	35,622 (51.0)	10,290 (14.7)
Female	6671 (12.5)	6852 (12.8)	37,868 (70.8)	2132 (4.0)	31,908 (20.8)	12,520 (8.2)	88,359 (57.8)	20,312 (13.3)
**Race/ethnicity**								
non-Hispanic White	8142 (11.8)	9799 (14.2)	48,174 (69.9)	2818 (4.1)	37,093 (19.4)	16,390 (8.6)	111,365 (58.2)	26,424 (13.8)
non-Hispanic Black	710 (16.8)	1345 (31.8)	2041 (48.2)	139 (3.3)	4808 (28.3)	2931 (17.3)	7188 (42.4)	2045 (12.0)
non-Hispanic Other	116 (18.2)	188 (29.4)	301 (47.1)	34 (5.3)	1290 (29.3)	715 (16.3)	1850 (42.1)	544 (12.4)
Hispanic	407 (24.9)	469 (28.7)	693 (42.4)	66 (4.0)	3611 (34.8)	1590 (15.3)	3578 (34.5)	1589 (15.3)
**Age group**								
65–74 years	839 (17.6)	1057 (22.2)	2665 (56.0)	194 (4.1)	2864 (23.6)	1666 (13.7)	5879 (48.5)	1717 (14.2)
75–84 years	3512 (14.2)	4502 (18.2)	15,641 (63.2)	1084 (4.4)	13,020 (22.6)	6567 (11.4)	29,872 (51.9)	8132 (14.1)
85 years and over	5024 (10.9)	6242 (13.6)	32,903 (71.6)	1779 (3.9)	30,918 (20.2)	13,393 (8.7)	88,230 (57.6)	20,753 (13.5)
**Marital status**								
Single	317 (7.8)	612 (15.1)	3008 (74.0)	128 (3.1)	1345 (13.3)	1159 (11.5)	6542 (64.6)	1076 (10.6)
Married	3466 (16.9)	4156 (20.3)	11,973 (58.5)	859 (4.2)	16,208 (27.7)	6822 (11.6)	27,109 (46.3)	8462 (14.4)
Widowed/Divorced	5592 (11.0)	7033 (13.8)	36,228 (71.1)	2070 (4.1)	29,249 (19.0)	13,645 (8.8)	90,330 (58.5)	21,064 (13.7)
**Education attainment**								
High School or Less	6645 (11.9)	8950 (16.0)	38,369 (68.5)	2038 (3.6)	31,052 (20.4)	15,231 (10.0)	86,124 (56.7)	19,598 (12.9)
Some College or College Degree	2159 (13.6)	2303 (14.5)	10,612 (66.8)	820 (5.2)	12,542 (21.8)	5142 (9.0)	30,933 (53.9)	8797 (15.3)
Advanced Degree	571 (16.1)	548 (15.5)	2228 (62.8)	199 (5.6)	3208 (23.6)	1253 (9.2)	6924 (50.9)	2207 (16.2)

Compared with 2000, rates of deaths at home and other places increased and rates of hospital and nursing home/long term care deaths decreased in 2014 across population cohorts defined by sex, race/ethnicity, age, marital status and education attainment. Moreover, the differences between population cohorts for the year 2000 persisted into 2014. Compared with males, females were still more likely to die at nursing home/long term care facility (Female: 57.8% vs. Male: 51.0%) and less likely to die at hospital (Female: 8.2% vs. Male: 13.0%). Non-Hispanic Whites were still much more likely to die at nursing home/long term care facilities and much less likely to die at their homes compared with other racial/ethnic groups; however, within the group, the difference between the proportions of nursing home/long term care deaths and home deaths diminished. Hispanics were equivalently split between home and nursing home/long term care deaths (decedent’s home: 34.8% vs. nursing home/long term care: 34.5%). The oldest age group (85 years and over) had the largest increase in the proportion of home deaths (from 10.9% in 2000 to 20.2% in 2014) and largest decrease in the proportion of nursing home/long term care facility deaths (from 71.6% in 2000 to 57.6% in 2014). Decedents who were married at death were still much more likely than their counterparts to die at home and much less likely to die at nursing home/long term care in 2014; those who were widowed/divorced had the largest decrease (from 71.1% in 2000 to 58.5% in 2014) in the proportion of nursing home/long term care deaths. And last, the decrease in the proportion of nursing home/long term care deaths was slightly more pronounced for decedents who had some college or college degree education; the increase in the proportion of home deaths was less pronounced for decedents who had advanced degree education.

### State variations

**Fig. 2.**
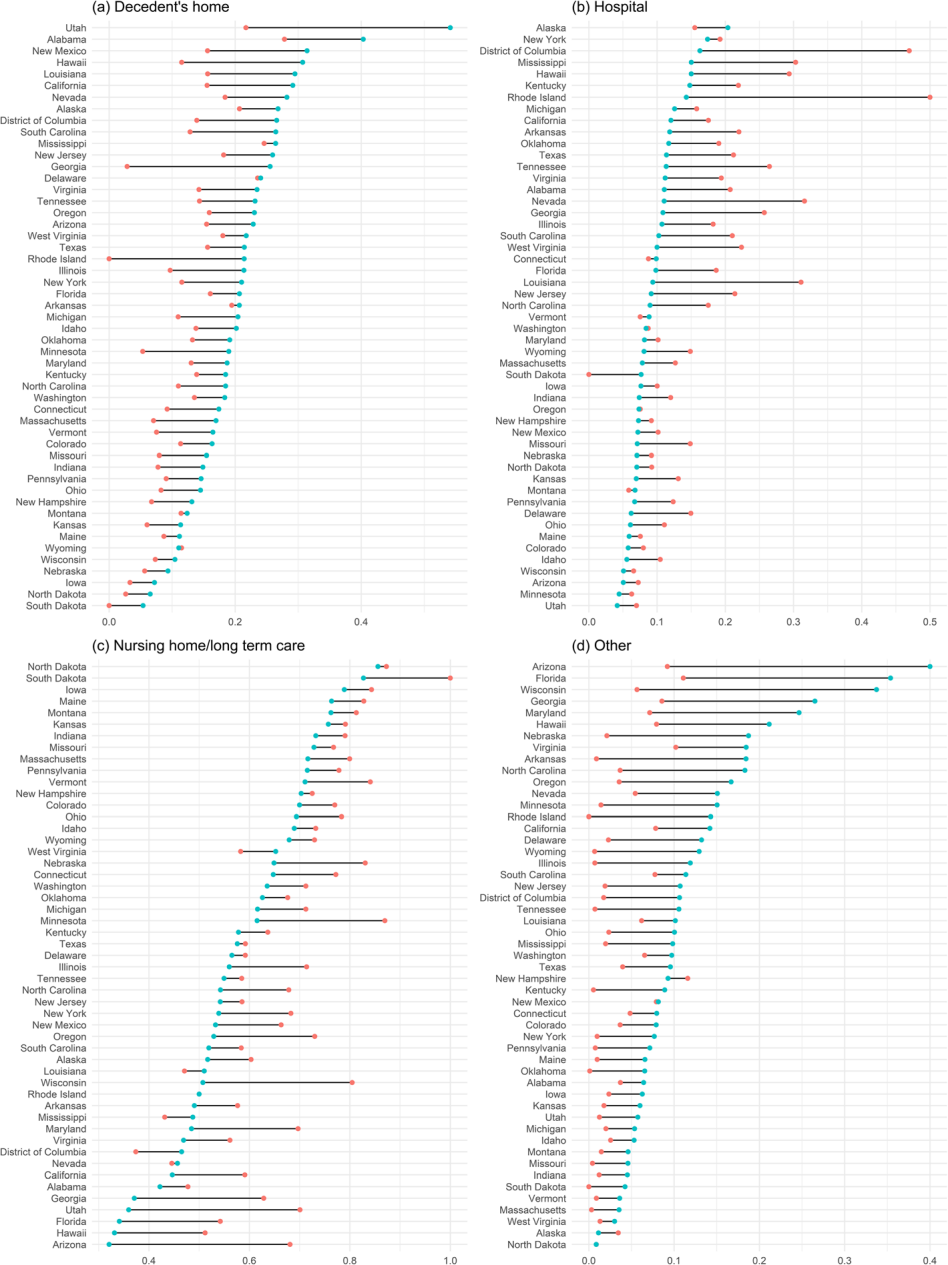
Inter-state and temporal variations in the proportion of dementia deaths at (**a**) Decedent’s home (**b**) Hospital (**c**) Nursing home/long term care (**d**) Other between 2000 and 2014. Red dots: 2000, blue dots: 2014

Figure 2 shows the state changes in the proportions of deaths at different places between 2000 and 2014 (Red dots represent the year 2000 and blue dots represent the year 2014. All 50 states and the District of Columbia are ordered by the proportion in 2014.). Utah had the highest proportion of home deaths (54.1%) in 2014 among all, followed by Alabama (40.3%), New Mexico (31.4%), Hawaii (30.7%), and Louisiana (29.5%). The states with the lowest proportion of home deaths in 2014 were South Dakota (5.4%), North Dakota (6.5%), Iowa (7.2%), Nebraska (9.3%), and Wisconsin (10.4%). Utah also had the largest increase in the proportion of home deaths among all states and the District of Columbia, from 21.7% in 2000 to 54.1% in 2014. For hospital deaths, the highest proportions in 2014 were in Alaska (20.4%), New York (17.4%), the District of Columbia (16.3%), Mississippi (15.0%), and Hawaii (15.0%) and the lowest were in Utah (4.2%), Minnesota (4.4%), Arizona (5.1%), Wisconsin (5.1%) and Idaho (5.6%). The largest decrease occurred in Rhode Island, from 50.0% in 2000 to 14.3% in 2014. For deaths at nursing home/long term care, states with the highest proportions in 2014 were North Dakota (85.6%), South Dakota (82.7%), Iowa (78.9%), Maine (76.4%), and Montana (76.2%); and the ones with the lowest proportions were Arizona (32.1%), Hawaii (33.1%), Florida (34.1%), Utah (36.0%), and Georgia (37.1%). The largest decease was in Arizona, from 68.1% in 2000 to 32.1% in 2014. Contradictory to the overall trend, a few states had an increase in the proportion of nursing home/long term care deaths during the study period, including West Virginia, Louisiana, Mississippi and the District of Columbia. For deaths at other places, the highest proportions in 2014 were in Arizona (40.0%), Florida (35.4%), Wisconsin (33.7%), Georgia (26.5%), and Maryland (24.6%) and the lowest were in North Dakota (1.0%), Alaska (1.1%), West Virginia (3.0%), Massachusetts (3.5%), and Vermont (3.6%). Arizona had the largest increase in the proportion of deaths at other places, from 9.2% in 2000 to 40.0% in 2014. It should be noted that a few states had very small numbers of deaths from dementia over the study period. For example, Rhode Island reported only 169 cases of dementia deaths (12 home deaths, 31 hospital deaths, 109 nursing home/long term care deaths, and 15 deaths at other places) during the 15-year study period. The state- and year-specific proportions of dementia deaths at a certain place of death generated from these smaller numbers are subject to greater variability. To ensure that parametric coefficient estimates in the regressions were more robust to the influence of the small numbers, states with the reported number of dementia deaths at any place in any year smaller than 30 were excluded from the panel data regression analysis.

### Associations between place of death and state level factors

The state fixed effects model results showing the associations between state-level factors and place of death resulting from a dementia-related disease are presented in Table 2. The independent variables explained 64%, 43%, and 48% of the variations in the proportions of home deaths, hospital deaths and nursing home/long term care deaths, respectively. To examine the possible multicollinearity among independent variables, a pooled panel data analysis was carried out (results not presented). The variation inflation factors (VIFs) of all independent variables were less than 5, indicating low levels of multicollinearity.

**Table 2:** Regression estimates from logit transformation of proportion panel data models with state fixed effects

Variable	Decedent’s home		Hospital		Nursing home / long term care	
	훃	(SE)	훃	(SE)	훃	(SE)
**Socio-demographic structure**						
% 85 years and older	0.51	(0.36)	−2.30***	(0.42)	−0.06	(0.47)
% Female	0.78	(0.50)	−1.83**	(0.60)	0.78	(0.66)
% non-Hispanic White	−2.20***	(0.47)	0.63	(0.56)	2.20***	(0.62)
% Married	1.84***	(0.55)	−1.09	(0.65)	−1.40	(0.72)
% High school or less	−1.85***	(0.40)	2.15***	(0.47)	2.00***	(0.52)
**Care facility resources**						
Hospital beds	−0.06	(0.05)	0.25***	(0.06)	−0.07	(0.06)
Nursing home beds	−0.02***	(0.00)	0.00	(0.00)	0.02***	(0.00)
**Public care financing**						
Medicare HHA	0.02	(0.07)	−0.44***	(0.08)	0.23*	(0.09)
Medicare HSNF	0.01	(0.02)	0.01	(0.02)	−0.04	(0.03)
Medicaid institutional LTSS	−0.12***	(0.03)	−0.06	(0.03)	0.17***	(0.04)
Medicaid HCBS	0.09***	(0.03)	0.05	(0.03)	−0.15***	(0.03)
Number of observations	645		645		645	
Number of groups	43		43		43	
*F*-statistics	111.1		49.8		58.6	
*p*-value	< 0.01		< 0.01		< 0.01	
Adj. *R*^*2*^	0.64		0.43		0.48	

Note: Alaska, District of Columbia, Georgia, South Dakota, North Dakota, Rhode Island, Vermont and Wyoming were excluded from analyses due to small numbers of death

*** p < 0.001, ** p < 0.01, * p < 0.05

The results showed that socio-demographic structure of dementia decedents was associated with death rates at different places at the state level. Specifically, the percentage of decedents who were married at the time of death was positively associated with the rates of home deaths (β=1.84, SE = 0.55, *p* < 0.001); while the percentages of decedents who were non-Hispanic White (β= − 2.20, SE = 0.47, *p* < 0.001) and who had an education of high school or less (β= − 1.85, SE = 0.40, *p* < 0.001) were negatively associated with rates of home deaths. The results also indicate a significant, positive association between rates of hospital deaths and the percentage of decedents who had an education of high school or less (β=2.15, SE = 0.47, *p* < 0.001). The percentage of female decedents (β= − 1.83, SE = 0.60, *p* < 0.01) and the percentage of decedents who were 85 years and older (β= − 2.30, SE = 0.42, *p* < 0.001), however, were negatively associated with rates of hospital deaths. Both the percentage of non-Hispanic White decedents (β=2.20, SE = 0.62, *p* < 0.001) and the percentage of decedents who had an education of high school or less (β=2.00, SE = 0.52, *p* < 0.001) were significantly, positively associated with rates of nursing home/long term care deaths.

Access to care facility resources also appeared to be associated with place of death among dementia patients. The number of hospital beds per 1000 population had a significant, positive association with the proportion of hospital deaths (β=0.25, SE = 0.06, *p* < 0.001); however, it was not significantly associated with rates of either home or nursing home/long term care deaths. The number of nursing home beds per 1000 older adults was negatively associated with home deaths (β= − 0.02, SE = 0.00, *p* < 0.001) and positively associated with nursing home/long term care deaths (β=0.02, SE = 0.05, *p* < 0.001).

Public financing of care, measured by Medicare and Medicaid investments in various health agencies/services, was also significantly associated with place of death of dementia decedents. Medicare reimbursement rate on HHA was positively associated with rates of nursing home/long term care deaths (β=0.23, SE = 0.09, *p* < 0.05) and negatively associated with rates of hospital deaths (β= − 0.44, SE = 0.08, *p* < 0.001); however, it was not significantly associated with rates of home deaths. Medicare reimbursement rate on HSNF was not related to either of the three outcomes. Medicaid expenditure rate on institutional LTSS was negatively associated with rates of home deaths (β= − 0.12, SE = 0.03, *p* < 0.001) and positively associated with rates of nursing home/long term care deaths (β=0.17, SE = 0.04, *p* < 0.001) but not hospital deaths. In the meantime, Medicaid expenditure rate on HCBS had a significant, negative association with nursing home/long term care deaths (β= − 0.15, SE = 0.03, *p* < 0.001) and a significant, positive association with rates of home deaths (β=0.09, SE = 0.03, *p* < 0.001).

Results from the two-way fixed effect models indicate that the direction, size and statistical significance of the coefficient estimates of most independent variables did not differ substantially from the one-way state fixed effects models, especially for care facility resources and public care financing variables (see Additional file 1). One notable difference is the relationship between availability of hospital beds and nursing home beds and proportion of hospital deaths. The number of hospital beds per 1000 population was not significantly associated with rates of hospital deaths after accounting for time fixed effects; however, the number of nursing home beds per 1000 older adults had a significant, negative association with rates of hospital deaths (β= − 0.01, SE = 0.00, *p* < 0.001).

## Discussion

This study empirically assessed changes over time in place of death among US older adults who reported dementia as the underlying cause of death between 2000 and 2014. Nationally, older adults who died from dementia were increasingly more likely to die at their homes, instead of institutional settings such as hospitals and nursing home/long term care facilities. The shares of deaths at the two institutional settings had both dwindled, although their combination still made up the majority. The share of deaths occurred at places other than the three mentioned above also increased during the study period, partly due to the increasing use of hospice facilities. Meanwhile, there was wide inter-state variability in where dementia decedents died as well as the extent to which the place of death of dementia decedents had changed between 2000 and 2014. Regression model diagnostics also indicated that large proportions of the variations in proportions of death at different places can be explained by our models. The largest changes, such as the increase in the proportion of home deaths in Utah and the decease in the proportion of nursing home/long term care deaths in Arizona, were likely related to changes in socio-demographic composition of dementia decedents, characteristics of local care facility provision and structures of public care financing.

Socio-demographic characteristics were associated with place of death of dementia decedents, as shown in Table 2. Both the percentage of decedents who were 85 years and older and the percentage of dementia decedents who were female showed a significantly negative association with rates of hospital deaths, after accounting for other covariates. However, these two variables did not show a significant association with either rate of home deaths or rate of nursing home deaths. Multiple studies have shown higher utilization of nursing homes and other institutional long term care services among Whites compared with racial and ethnic minorities [46–51]. Race and ethnicity represent life-long socioeconomic status that can contribute to differential patterns of end-of-life care, of which value is a decisive factor [15]. The percentage of decedents who were married was positively associated with rates of home deaths, demonstrating the significant role of spousal social support in avoiding institutional placement. The percentage of decedents who had high school or less education showed a positive relationship with rates of institutional deaths and a negative relationship with rates of home deaths. This may be because that education attainment, on average a quality indicator of life-long socioeconomic status, increases access to information, power and resource, and subsequently gives patients and their families latitude to make end-of-life care decisions that are more in line with their preferences. Interestingly, the relationship between education and institutional/home death appeared to be in the opposite direction from the one between race/ethnicity and institutional/home death. It points to the complexity of race and ethnicity as social constructs with a range of social meanings that may influence where dementia patients choose to die. For example, studies have shown that race and ethnicity are related to the access to long term care/end-of-life care services [52–54] and the cultural norms and attitudes towards these services (e.g. spirituality, family structure, language barriers, mistrust of the systems) [47, 55–57].

One of the aims of this study was to relate changes in place of death of older adults who died from dementia to the state provisions of care facilities and finance resources. Results showed that the availability of care facility resources was directly associated with rates of deaths at that facility. Larger state availability of nursing home beds was associated with higher rates of deaths in nursing home/long term care facilities; so was the case for hospital beds and deaths at hospitals. States have been implementing various policies to control the provider supply for institutional long term care over the last a few decades. One major strategy is through the certificate-of-need (CON) programs. In 1974, the National Health Planning and Resources Development Act (P.L. 93–641) prescribed that states must have structures involving submission of proposals and gaining the approval of certificate-of-need regulators before starting any capital projects including establishing new health care facilities and providing new services [58, 59]. Although the federal government did not reauthorize the national CON requirements in 1987, many states continued their CON programs under state legislative authority [58]. As of 2016, 35 states and the District of Columbia maintain some form of CON program, 12 states have discontinued their CON programs and 3 states have some variations [59] (see Additional file 2 for state CON law status and if certain facilities are regulated). Although the facilities and services regulated by CON laws vary widely from state to state, current CON laws are more regulatory towards long term care and outpatient facilities [60]. Some states additionally introduced moratoria programs to strengthen their regulation on provider supply. Partly due to these regulatory policies, the nursing home beds availability had steadily decreased across all states (see Additional file 3), potentially contributing to the decreased proportions of dementia deaths at nursing home/long term care settings. Meanwhile, the availability of hospital beds also appeared to be more limited over time in most states (see Additional file 4).

Long term care is expensive. Care for patients with dementia is much more costly than for patients with other diseases [61]. Also, costs of care delivered at institutional settings are significantly higher than care at home [62, 63]. Federal and state investments in public insurance programs such as Medicare and Medicaid can significantly impact patient’s purchase power of end-of-life care at different settings and can consequently influence their place of death. Our results showed that more generous Medicaid expenditure on institutional LTSS was associated with higher proportion of nursing home/long term care deaths and lower proportion of home deaths. States with higher investments in institutional LTSS may provide an incentive for care facilities such as nursing homes to keep providing services to patients instead of transferring them to hospitals [15, 64, 65]. Also, nursing facilities in states with higher Medicaid nursing home reimbursement rates were more likely to hire nurse practitioners and physician assistants [66]. Better quality of care due to improved staffing may propel patients and their relatives to make the decision to enter nursing facilities and stay. As for HCBS, increased Medicaid expenditure was associated with higher rates of home deaths and lower rates of nursing home/long term care deaths. Reasonably, dementia patients with better support at home and in the community would be better equipped to avoid institutional placement. There was a symmetrical relationship between institutional LTSS spending/dying at nursing home/long term care and HCBS spending/dying at home. Medicaid expenditure, however, was not significantly associated with the rates of hospital deaths (For temporal trends in state Medicaid expenditure on institutional LTSS and HCBS, see Additional files 5 and 6, respectively).

Medicare benefit spending was also associated with place of death of dementia decedents. Specifically, more generous Medicare reimbursement rate on HHA was negatively associated with the rates of hospital deaths and positively associated with rates of nursing home/long term care deaths. Surprisingly, Medicare reimbursement rate on HHA was not significantly associated with rates of home deaths. Medicare covers home health care for persons who are homebound and in need of part-time nursing care or therapy services [67]. Medicare expenditure on HSNF was not associated with rates of deaths at either of the three places. This may be because Medicare is not designed to pay for long term care. Medicare only pays for skilled nursing facilities for up to 100 days following a hospital stay of at least 3 consecutive days (postacute care) and offers hospice care benefits at the end of life [67–69]. For most dementia patients who usually live a much longer period of time, Medicare is not the primary source of finance for long term care (For temporal trends in state Medicare reimbursement rates on HHA and HSNF, see Additional files 7 and 8, respectively).

This study has several limitations, too. First, several states had very small numbers of dementia deaths across different places. Those states were excluded from the panel data analysis due to the potential biases that could be introduced to parametric estimates. The relationships found between place of death and state factors may not be generalized to those less populated states. Where dementia patients die in those rural states and how it is related to sociodemographic features, access to care facilities, and state financial supports may warrant special attention. Second, this study examined the relationships between state care financing, specifically Medicare reimbursements on HHA and HSNF and Medicaid expenditure on institutional LTSS and HCBS, and place of death among dementia patients. However, how much of those expenditures was directed towards older adult dementia patients is not clear. To better inform policies designed to face the challenge of providing quality end-of-life care for an increasing number of dementia patients, federal and state agencies need to create a data taxonomy that is more reflective of service use and spending of this sub-population. Additionally, the care finance factors in this study were focused on public expenditures. Other market characteristics such as out-of-pocket spending and financing through private long term care insurance were not examined. These factors might also have had a large impact on where dementia patients die because they make up a significant portion of long term care payment [70]. And lastly, transition between places of care for dementia patients at the end of their lives is not uncommon. It is entirely possible that a dementia patient received end-of-life care at home or nursing home but was transferred to a hospital during episodes of acute complications of dementia and died there. End of life experiences of dementia patients vary widely and their implication for the place of death needs much deeper examination.

## Conclusion

Despite that the national rates of home deaths among dementia decedents had increased over time, the majority still died elsewhere. The rates of home deaths, as well as the extent to which the rates changed over time, vary substantially among states. Our results also indicate that both lower availability of nursing home beds and greater Medicaid expenditure on home and community based services were significantly associated with higher rates of home deaths among dementia decedents at the state level. Given the uncertain externalities of limiting nursing home resources, our findings imply that expanding expenditures on home and community based services may be an effective way to help dementia patients to die at their homes.

## Supplementary information

**Additional file 1.** Associations between state-level factors and place of death of dementia decedents (two-way fixed effects models)

**Additional file 2.** State status of Certificate of Need (CON) laws and whether certain facilities were regulated

**Additional file 3.** Temporal trend in nursing home bed availability by state, 2000–2014

**Additional file 4.** Temporal trend in hospital bed availability by state, 2000–2014

**Additional file 5.** Temporal trend in Medicaid expenditure on institutional LTSS by state, 2000–2014

**Additional file 6.** Temporal trend in Medicaid expenditure on HCBS by state, 2000–2014

**Additional file 7.** Temporal trend in Medicare reimbursement rate on HHA by state, 2000–2014

**Additional file 8.** Temporal trend in Medicare reimbursement rate on HSNF by state, 2000–2014

## Data Availability

The dataset supporting the conclusions of this article are available from the National Center for Health Statistics. Restrictions apply to the availability of these data, which were used under license for the current study.

## Abbreviations

AD: Alzheimer’s disease
CMHS: Continuous Medicare History Sample
CMS: Centers for Medicare and Medicaid Services
CON: Certificate of need
HB: Hospital beds
HCBS: Home and community based services
HHA: Home health agency
HSNF: Hospital and skilled nursing facility
ICD: International Classification of Diseases
LTSS: Long term services and support
NCHS: National Center for Health Statistics
NHB: Nursing home beds
US: United States
VIF: Variation Inflation Factor

## Footnotes

1 We first combined the original *other* and *place of death unknown* because both categories are “black boxes” and provide little information of decedents’ actual place of death. For *hospice facility*, the National Center for Health Statistics started to record it as a stand-alone *Place of death* category on death certificates in 2003. In that year, only 178 dementia deaths (1.8‰ of all dementia deaths) occurred at hospice facilities in the United States and were concentrated in 5 states (Arkansas, California, Idaho, Montana, and New York). Moreover, according to the National Hospice and Palliative Care Organization, only 1.3% of days of hospice care were provided at hospice inpatient facility in 2016 (see https://www.nhpco.org/sites/default/files/public/Statistics_Research/2017_Facts_Figures.pdf). Using *hospice facility* as a *Place of death* category does not reflect the actual use of hospice care among dementia patients. Thereby, *hospice facility* was also combined into the *Other* category.

2 CMHS-based Medicare reimbursement rate was calculated from 5% sample of Medicare beneficiaries provided in the Continuous Medicare History Sample (CMHS) file. As Medicare payment method shifted toward claim-based, the CMHS-based method was discontinued by the Centers for Medicare and Medicaid Services (CMS). The Dartmouth Atlas Project provides state CMHS-based Medicare reimbursement rates for the years 1992–2007 and started calculating claim-based reimbursement rates in 2003 (discussions of the differences between the 5% CMHS sample and the 20% claim sample can be found at https://atlasdata.dartmouth.edu/pdf/PA_Spending_Report_0611.pdf). As the website states, “The availability of comprehensive claims files and the greater number of patients represented in a 20% sample (20% of patients included in the CMS physician/Supplier Part B file) have made it feasible to calculate per capita spending directly from the claims data.” By comparing the 2007 CMHS-based sample and claims-based sample, the researchers found that the per capita Medicare spending, as well as spending growth rates, calculated from the two samples were highly correlated at the hospital referral regions (HRR) level. They conclude that it is acceptable to “chain” the two datasets to construct a dataset covering a longer time period. In this study, data for years 2000–2003 were based on CMHS files and data for years 2004 and afterward were claims-based.
