# Supplementary material for: Assessment of changes in place of death of older adults who died from dementia in the United States, 2000–2014: a time-series cross-sectional analysis
Source: BMC Public Health. 2020 Jun 11;20:765. doi: 10.1186/s12889-020-08894-0 (PMC7288493; doi:10.1186/s12889-020-08894-0)
Supplement: Supplementary file 1 — Additional file 1. Associations between state-level factors and place of death of dementia decedents (two-way fixed effects models) [file 12889_2020_8894_MOESM1_ESM.docx]

Associations between state-level factors and place of death of dementia decedents (two-way fixed effects models).

| **Variables** | **Decedent's home** | |  | **Hospital** | |  | **Nursing home / long term care** | |
| --- | --- | --- | --- | --- | --- | --- | --- | --- |
|  | 𝛃 | (SE) |  | 𝛃 | (SE) |  | 𝛃 | (SE) |
| **Socio-demographic structure** |  |  |  |  |  |  |  |  |
| % 85 years and older | -0.90* | (0.43) |  | -0.86 | (0.47) |  | 0.48 | (0.58) |
| % female | 1.34** | (0.50) |  | -2.58*** | (0.55) |  | 0.76 | (0.68) |
| % non-Hispanic White | -1.57** | (0.48) |  | -0.44 | (0.52) |  | 2.27*** | (0.65) |
| % married | 1.58** | (0.55) |  | -1.22* | (0.60) |  | -1.06 | (0.74) |
| % high school or less | -0.84* | (0.42) |  | 0.22 | (0.46) |  | 2.08*** | (0.57) |
| **Care facility resources** |  |  |  |  |  |  |  |  |
| Hospital beds | 0.04 | (0.05) |  | 0.06 | (0.05) |  | -0.07 | (0.07) |
| Nursing home beds | -0.01*** | (0.00) |  | -0.01*** | (0.00) |  | 0.02*** | (0.00) |
| **Public care financing** |  |  |  |  |  |  |  |  |
| Medicare HHA | -0.07 | (0.07) |  | -0.43*** | (0.08) |  | 0.31** | (0.10) |
| Medicare HSNF | -0.03 | (0.03) |  | -0.05 | (0.03) |  | 0 | (0.04) |
| Medicaid institutional LTSS | -0.13*** | (0.03) |  | -0.03 | (0.03) |  | 0.17*** | (0.04) |
| Medicaid HCBS | 0.06* | (0.03) |  | 0.04 | (0.03) |  | -0.12*** | (0.03) |
| Number of observations | 645 |  |  | 645 |  |  | 645 |  |
| Number of groups | 43 |  |  | 43 |  |  | 43 |  |
| *F*-statistics | 9.4 |  |  | 9.2 |  |  | 12.5 |  |
| *p*-value | <0.01 |  |  | <0.01 |  |  | <0.01 |  |
| Adj. R-Squared | 0.05 |  |  | 0.05 |  |  | 0.1 |  |

Note: Alaska, District of Columbia, Georgia, South Dakota, North Dakota, Rhode Island, Vermont and Wyoming were excluded from analyses due to small numbers of death. There are significant time-fixed effects for Decedent's home and Hospital outcomes, but not for Nursing home/ long term care. **** p<*0.001*, ** p<*0.01*, * p<*0.05
