# Supplementary material for: Assessment of changes in place of death of older adults who died from dementia in the United States, 2000–2014: a time-series cross-sectional analysis
Source: BMC Public Health. 2020 Jun 11;20:765. doi: 10.1186/s12889-020-08894-0 (PMC7288493; doi:10.1186/s12889-020-08894-0)
Supplement: Supplementary file 2 — Additional file 2. State status of Certificate of Need (CON) laws and whether certain facilities were regulated [file 12889_2020_8894_MOESM2_ESM.docx]

State Certificate of Need (CON) laws and facilities regulated.

| **State** | **Has CON law as of 2016?** | **Facility regulated** | | |
| --- | --- | --- | --- | --- |
|  |  | **Nursing home (beds)** | **Hospital (beds)** | **Home health agencies** |
| Alabama | yes | yes | yes | yes |
| Alaska | yes | yes | yes | no |
| Arizona | no | no | no | no |
| Arkansas | yes | yes | no | yes |
| California | no | no | no | no |
| Colorado | no | no | no | no |
| Connecticut | yes | yes | yes | no |
| Delaware | yes | yes | yes | no |
| District of Columbia | yes | yes | yes | yes |
| Florida | yes | yes | yes | no |
| Georgia | yes | yes | yes | yes |
| Hawaii | yes | yes | yes | yes |
| Idaho | no | no | no | no |
| Illinois | yes | yes | yes | no |
| Indiana | no | no | no | no |
| Iowa | yes | yes | yes | no |
| Kansas | no | no | no | no |
| Kentucky | yes | yes | yes | yes |
| Louisiana | yes | yes | no | yes |
| Maine | yes | yes | yes | no |
| Maryland | yes | no | yes | yes |
| Massachusetts | yes | yes | yes | no |
| Michigan | yes | yes | yes | no |
| Minnesota | no | yes | yes | no |
| Mississippi | yes | yes | yes | yes |
| Missouri | yes | yes | yes | no |
| Montana | yes | yes | yes | yes |
| Nebraska | yes | yes | no | no |
| Nevada | yes | yes | yes | yes |
| New Hampshire | no | no | no | no |
| New Jersey | yes | yes | yes | yes |
| New Mexico | no | no | no | no |
| New York | yes | yes | yes | yes |
| North Carolina | yes | yes | yes | yes |
| North Dakota | no | no | no | no |
| Ohio | yes | yes | no | no |
| Oklahoma | yes | yes | no | no |
| Oregon | yes | yes | yes | no |
| Pennsylvania | no | no | no | no |
| Rhode Island | yes | yes | yes | yes |
| South Carolina | yes | yes | yes | no |
| South Dakota | no | no | no | no |
| Tennessee | yes | yes | yes | yes |
| Texas | no | no | no | no |
| Utah | no | no | no | no |
| Vermont | yes | yes | yes | yes |
| Virginia | yes | yes | yes | no |
| Washington | yes | yes | yes | no |
| West Virginia | yes | no | yes | yes |
| Wisconsin | no | no | no | no |
| Wyoming | no | no | no | no |

Note: 1. Minnesota ended its CON programs in 1984 but started to implement a similar process called "public interest review" in 2004 to require proposal for constructing new hospital or hospital bed expansions. It also allows for exceptions to the moratorium on nursing homes. 2. New Hampshire ended its CON program in 2016. 3. Wisconsin maintains an approval process for nursing homes.
