# Supplementary figures and images for: Assessment of changes in place of death of older adults who died from dementia in the United States, 2000–2014: a time-series cross-sectional analysis
Source: BMC Public Health. 2020 Jun 11;20:765. doi: 10.1186/s12889-020-08894-0 (PMC7288493; doi:10.1186/s12889-020-08894-0)

# Trend in hospital bed availability, 2000–2014

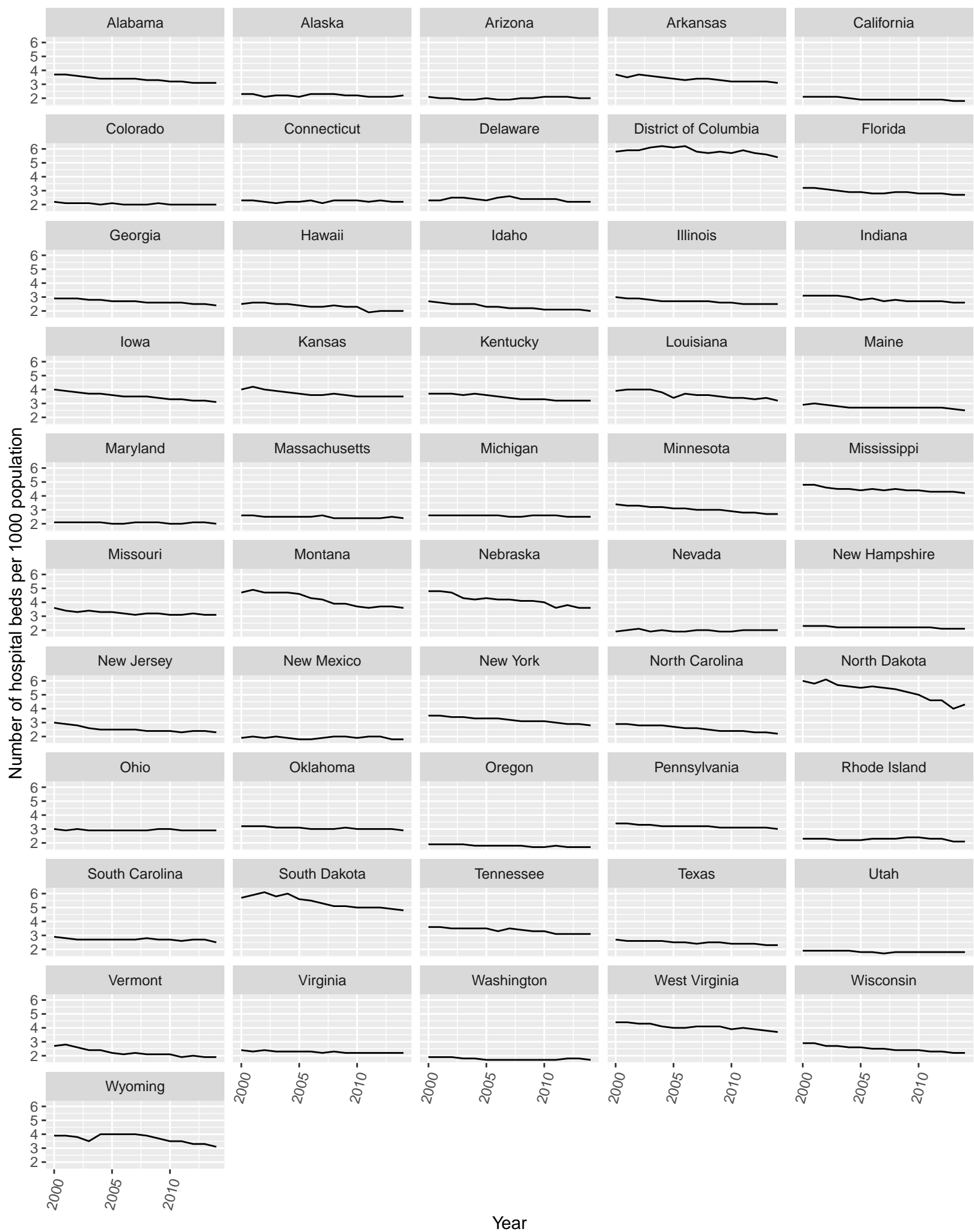

Supplement: Supplementary file 3 — Additional file 3. Temporal trend in nursing home bed availability by state, 2000–2014 [file 12889_2020_8894_MOESM3_ESM.pdf]

Trend in nursing home bed availability, 2000–2014

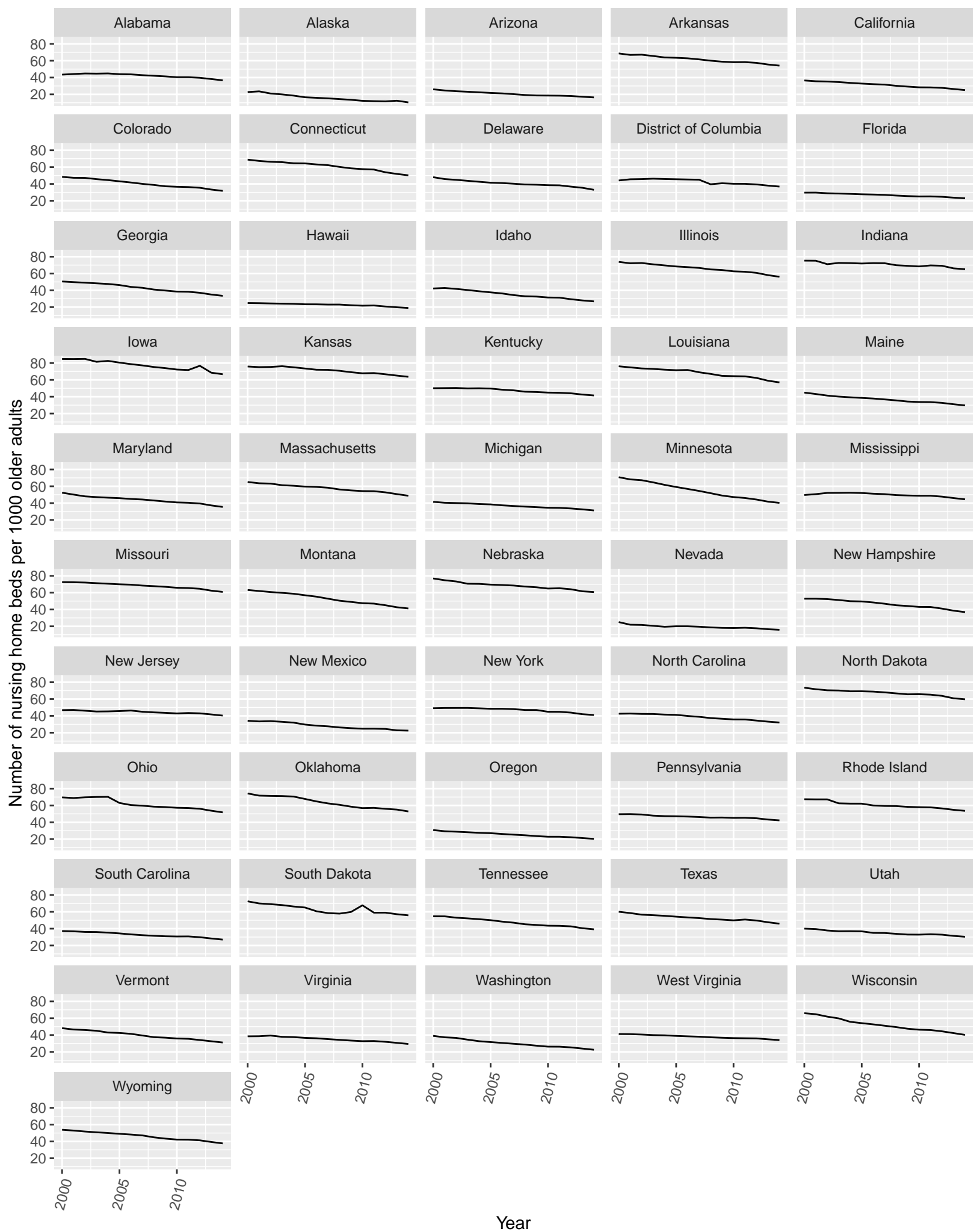

Supplement: Supplementary file 4 — Additional file 4. Temporal trend in hospital bed availability by state, 2000–2014 [file 12889_2020_8894_MOESM4_ESM.pdf]

Trend in Medicaid expenditure on institutional LTSS, 2000–2014

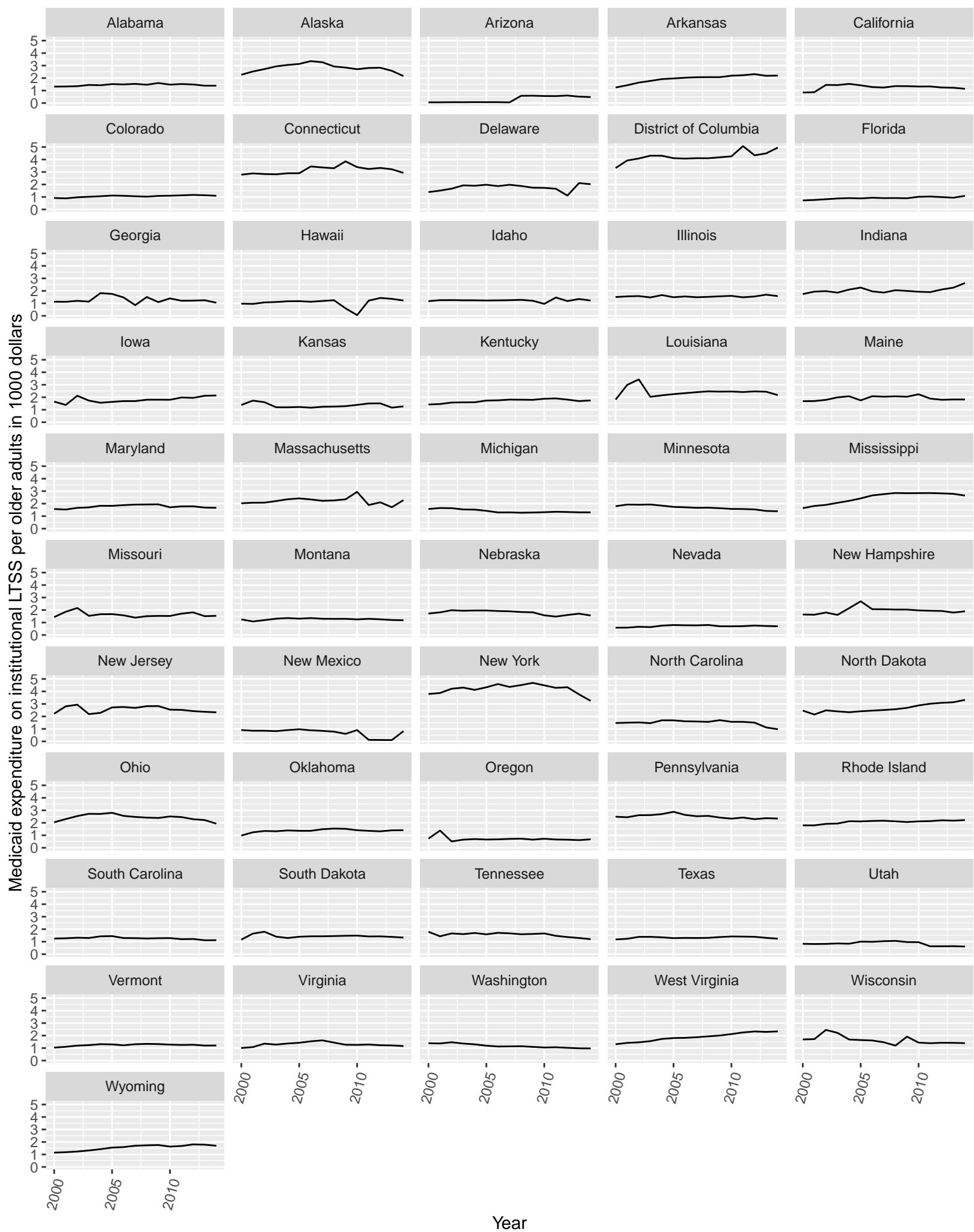

Supplement: Supplementary file 5 — Additional file 5. Temporal trend in Medicaid expenditure on institutional LTSS by state, 2000–2014 [file 12889_2020_8894_MOESM5_ESM.pdf]

Trend in Medicaid expenditure on HCBS, 2000–2014

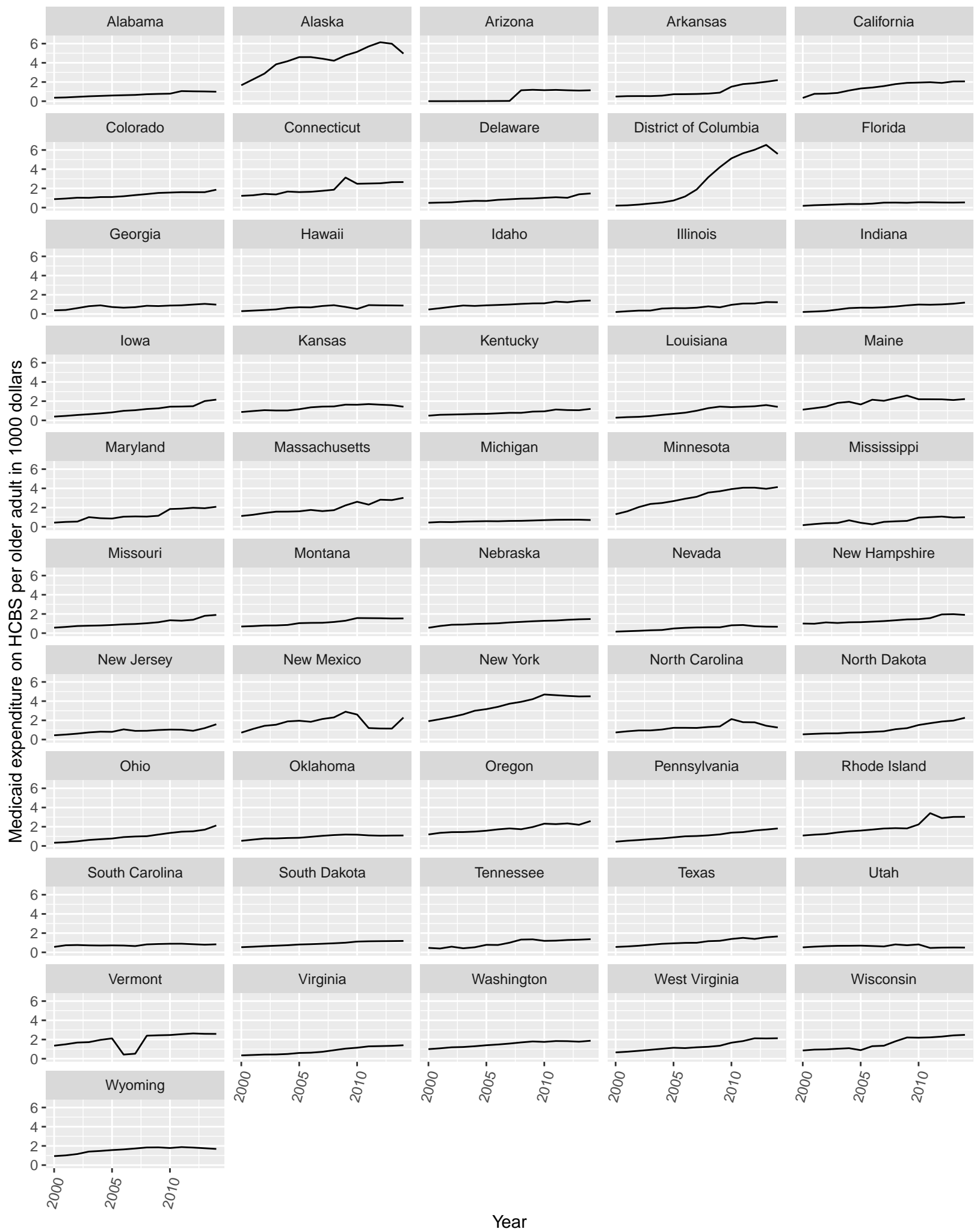

Supplement: Supplementary file 6 — Additional file 6. Temporal trend in Medicaid expenditure on HCBS by state, 2000–2014 [file 12889_2020_8894_MOESM6_ESM.pdf]

Trend in Medicare reimbursement rate on HHA, 2000–2014

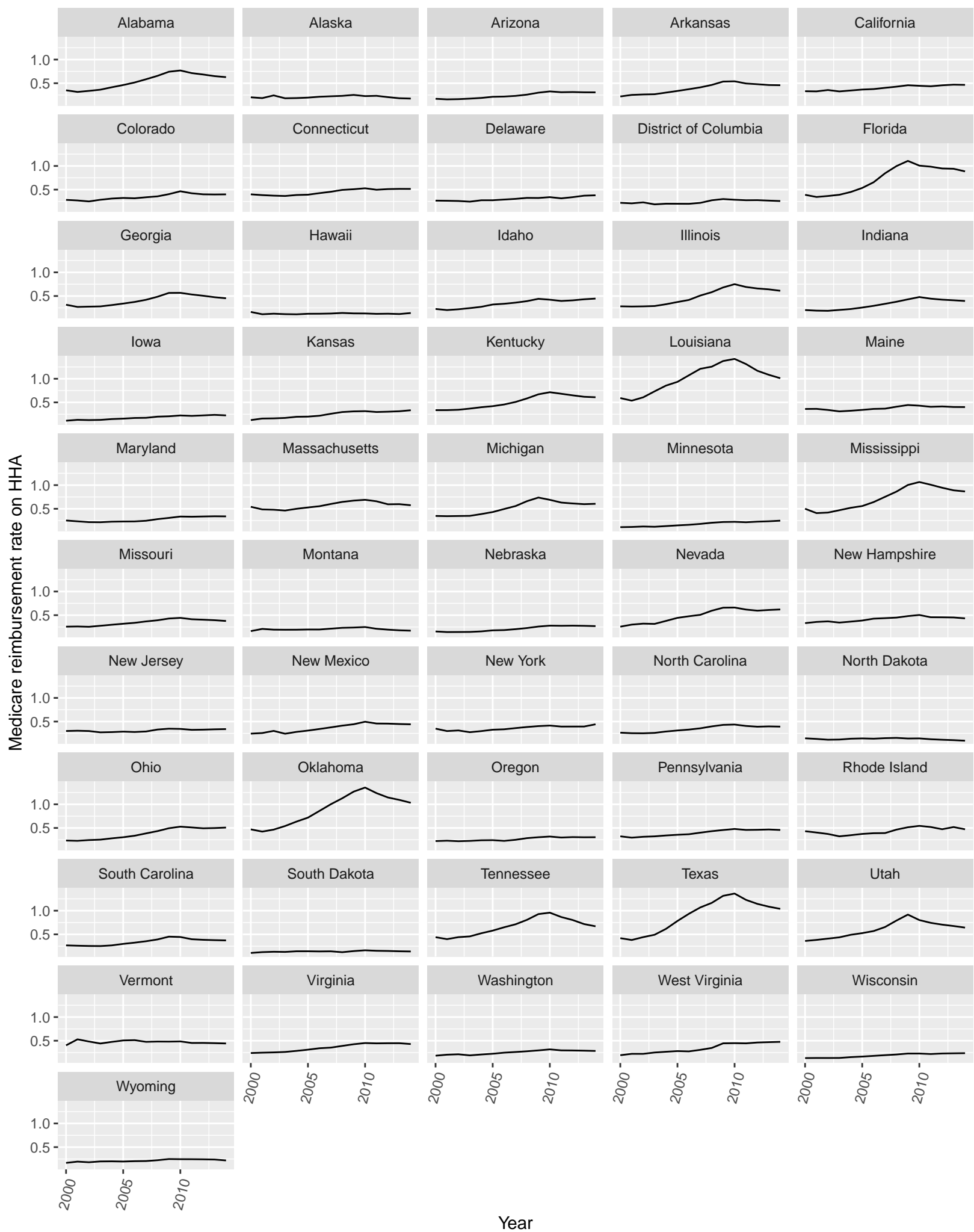

Supplement: Supplementary file 7 — Additional file 7. Temporal trend in Medicare reimbursement rate on HHA by state, 2000–2014 [file 12889_2020_8894_MOESM7_ESM.pdf]

Trend in Medicare reimbursement rate on HSNF, 2000–2014

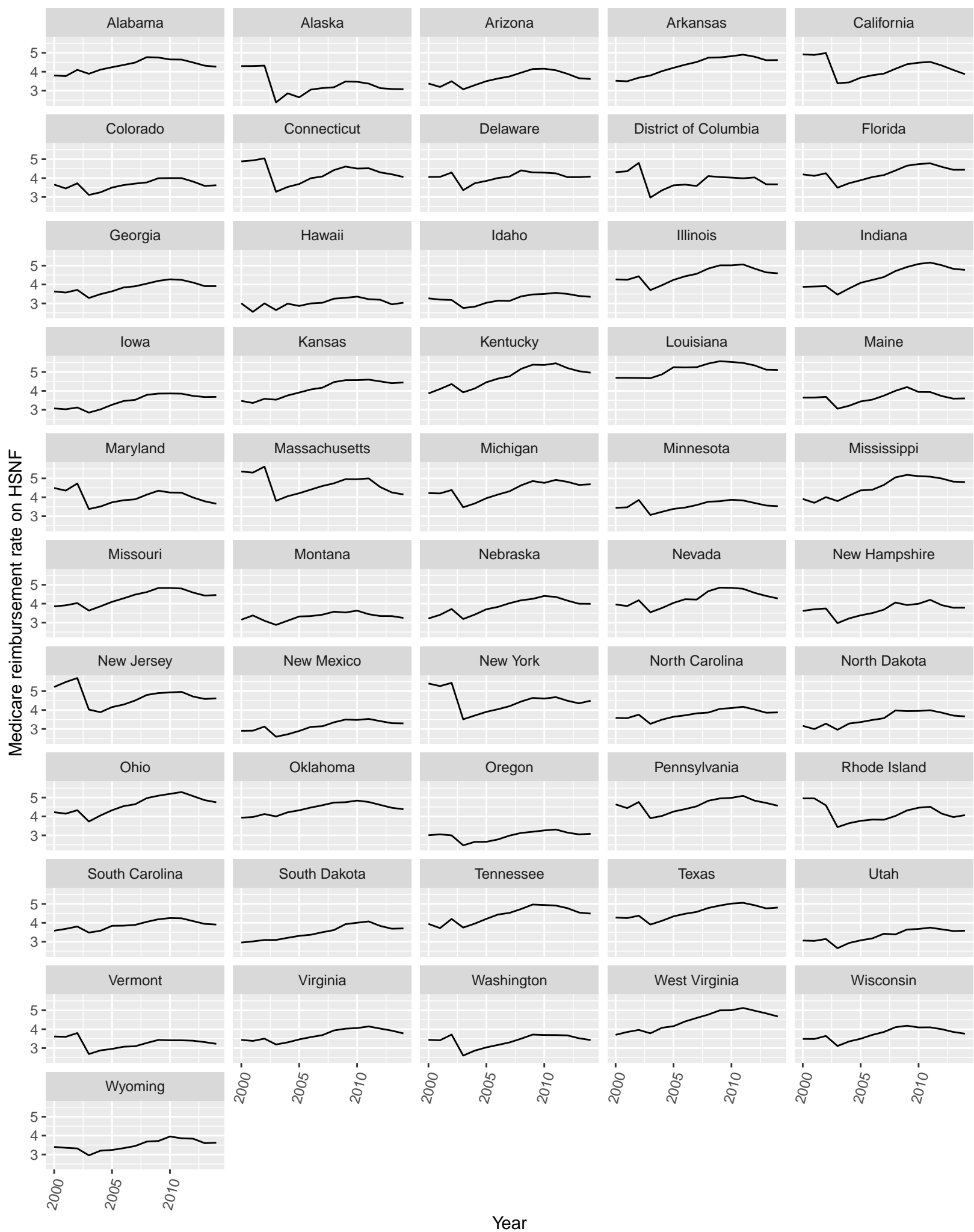

Supplement: Supplementary file 8 — Additional file 8. Temporal trend in Medicare reimbursement rate on HSNF by state, 2000–2014 [file 12889_2020_8894_MOESM8_ESM.pdf]
